# *Elymus* (Poaceae) as a model for climate-resilient crop design

**DOI:** 10.3389/fpls.2026.1740805

**Published:** 2026-02-06

**Authors:** Shuguang Yu, Tsechoe Dorji

**Affiliations:** 1 Institute of Tibetan Plateau Research, Chinese Academy of Sciences, Beijing, China; 2 University of Chinese Academy of Sciences, Beijing, China; 3 National Field Scientific Observation and Research Station of Alpine Grassland in Nagqu, Nagqu, China

**Keywords:** climate resilience, *de novo* domestication, *Elymus*, negotiated genome, sustainable agriculture

## Abstract

The escalating climate crisis demands that agriculture move beyond the incremental improvement of domesticated crops. We posit that the wild perennial grass genus *Elymus* offers a novel and powerful model for this transition. By synthesizing recent genomic, ecological, and physiological evidence, we show that the evolutionary success of these complex polyploids rests on three interconnected foundational principles: a dynamic “negotiated” allopolyploid genome balancing structural innovation with meiotic stability; a modular toolkit of molecular, microbial, and epigenetic components orchestrating stress resilience; and keystone ecosystem engineering functions enhancing soil health and carbon sequestration. Decoding this integrated “*Elymus* Model” provides a transformative framework that shifts the paradigm from transferring isolated traits to the principled design of crops and agroecosystems whose inherent multi-scale architecture confers productivity, sustainability, and climate resilience.

## Introduction

Global agriculture faces the twin challenges of shrinking arable land and increasingly volatile climates. The integrative framework of genomics-assisted breeding (GAB 2.0) for designing future crops (Varshney et al., 2021) provides a strategic path forward, emphasizing the targeted exploitation of pre-adapted genetic variation from wild relatives to overcome yield plateaus and enhance stress resilience. The grass genus *Elymus* (wheatgrass), which comprises predominantly perennial species with a broad ecological amplitude across global temperate regions, provides a powerful natural model for this pursuit. While extensively studied in demanding environments such as the Qinghai-Tibet Plateau (QTP), its adaptive strategies are representative of those found in congeners from saline, arid, and boreal habitats worldwide.

As genomically complex allopolyploids, *Elymus* species demonstrate what we term a dynamic “negotiated genome”—an ongoing state of genomic and epigenetic adjustment. This conceptualization builds upon the understanding that polyploid genomes undergo dynamic reorganization to balance evolutionary innovation with meiotic stability (Van De Peer et al., 2017). This dynamism generates the genetic and epigenetic diversity that facilitates rapid local adaptation (Li et al., 2024b). Crucially, this diversity is organized into a modular toolkit that comprises discrete, stress-responsive molecular, microbial, and epigenetic components that can be selectively recruited and assembled to counter specific challenges (Trivedi et al., 2020; Shilpa et al., 2024). Furthermore, the integrated expression of these adaptive modules often extends into keystone ecosystem functions, such as soil structure enhancement and beneficial microbiome recruitment, which in turn modify the plant’s environment.

We argue that the unique value of *Elymus* as a model lies in the hierarchical interplay of these three principles: the negotiated genome provides the raw material for variation, modularity governs the phenotypic assembly of this variation, and ecosystem engineering represents its functional, environmental expression. Together, they form a coherent “*Elymus* Model” of integrated adaptation. Decoding this model provides a novel framework for climate-resilient agriculture. Unlike approaches that focus solely on singular stress traits, this model provides a systems-level understanding. It moves the field beyond the transfer of isolated traits towards the principled design of crops and agroecosystems that are resilient through their integrated architecture. This review critically synthesizes the evidence underpinning the *Elymus* Model, explicitly distinguishing between well-supported mechanisms and emerging hypotheses for each pillar. We then chart the translational pathways this model unlocks for the design of future climate-resilient crops.

## The negotiated genome

Polyploidy is a dynamic evolutionary process that generates genomic novelty beyond mere duplication (Van De Peer et al., 2017). In *Elymus*, this is evidenced by widespread chromosomal rearrangements—including species-specific translocations and inversions—following allopolyploid formation (Liu et al., 2023a). A key mechanism for stabilizing these nascent polyploids involves such structural variations (SVs), which suppress recombination to protect co-adapted gene complexes. This function aligns with chromosomal speciation mechanisms (Rieseberg, 2001) and represents a critical factor in allopolyploid genome stabilization (Comai, 2005).

The recent generation of high-quality, chromosome-scale genome assemblies—such as that of *Elymus sibiricus* (*E. sibiricus*) cultivar Gaomu No.1 (Shen et al., 2024), which achieved robust phasing of its St and H subgenomes using subgenome-specific repetitive sequences and kmers—now enables deeper investigation into subgenome coordination and epigenomic regulation. We propose the “negotiated genome” as a framework to analyze how allopolyploid genomes balance innovation with stability, and to distinguish processes supported by direct evidence from those that remain hypothetical. The role of SVs, such as species-specific reciprocal translocations (e.g., 4H/6H in *E. sibiricus*), in stabilizing polyploid genomes is a well-supported mechanism. In contrast, the precise molecular processes of subgenome crosstalk (e.g., via small RNAs or protein interactions) and epigenetic regulation (e.g., DNA methylation reprogramming) are active hypotheses: current supporting evidence, such as differential gene expression between subgenomes, is correlative and awaits functional validation through approaches like single-cell RNA sequencing (scRNA-seq) combined with clustered regularly interspaced short palindromic repeats (CRISPR)-based editing. While epigenetic regulation is broadly implicated in plant genome stability (Leech et al., 2025), direct functional demonstration of its causal role in *Elymus* remains elusive.

Thus, research in *Elymus* productively integrates established mechanisms (e.g., SV-mediated stabilization) with frontier questions about subgenome coordination. The genus is a powerful model for this because its extensively documented SVs—from the QTP to other global regions—are coupled with frequent hybridization. Deciphering these processes can provide a blueprint for harnessing genomic plasticity in crop improvement.

### Structural variation as an adaptive driver

Building on the concept of the negotiated genome, SV is a major source of adaptive diversity underpinning local adaptation in *Elymus*. Cytogenetically confirmed intergenomic translocations provide direct evidence for this role: for example, whole-arm translocations involving chromosome 3H and translocated segments on 6St/7St are linked to cold tolerance in the northeastern Chinese accession *E. sibiricus* 20HSC-Z9 (Liu et al., 2024), while a newly identified 2H/5Y translocation in *E. dahuricus* represents a species-specific structural variant contributing to adaptation to high-altitude, cold, and arid habitats (Jiang et al., 2023). SV formation is not a historical event but an ongoing process, as demonstrated by the continuous emergence of novel translocations within hybrid swarms of *E. hispidus* and *E. repens* in Central Europe (Urfusová et al., 2024). Collectively, these examples across Eurasia—spanning Northeast Asia, the QTP, and Central Europe—confirm that adaptive SV is a widespread phenomenon across *Elymus*’ global range. Comparative studies further validate that chromosomal restructuring is common across the genus—often initiated by meiotic errors (Liu et al., 2022a, b)—functionally linking meiotic instability to the generation of adaptive novelty (Liu et al., 2023a).

Environmental selection plays a significant role in shaping SV landscapes. Population genetic analyses of *E. breviaristatus* show that isolation-by-environment can be a stronger driver of population structure than isolation-by-distance (Li et al., 2022), implying environmental selection acts on genomic SV. On an evolutionary scale, species divergence events in *E. sibiricus* correlate with major geological changes, such as the QTP uplift during the Himalayan Motion stage (Han et al., 2022). Environmental gradients leave direct imprints on genomic features: altitudinal clines of genome size in three *Elymus* species pinpoint mid-elevation zones (3400–3900 m) as hotspots of genomic variation (Chen et al., 2022). Putative adaptive signals have even been identified in organellar genomes—for example, unique haplotype composition and elevated transposon content in the mitochondrial genome of *E. sibiricus* are linked to high-altitude adaptation (Xiong et al., 2022b). A critical distinction must be made between correlation and mechanism. While these studies establish robust correlative links between environment and SV patterns, functional proof of how specific environmental pressures directly trigger or select for particular SVs is lacking. Establishing these causal mechanisms is a key frontier for understanding the negotiated genome framework.

To translate insights on SV into predictive power for climate change responses, research must address three key gaps. First, integrated pan-genome analyses are needed to pinpoint the molecular drivers of adaptive SVs—such as transposable element (TE) activity, which has been linked to high-altitude adaptation in *E. sibiricus* (Xiong et al., 2022a). Second, longitudinal monitoring of SV dynamics in natural hybrid zones across diverse ecosystems is essential. Baseline studies documenting widespread hybridization and recurrent chromosomal rearrangements in *Elymus* complexes (Urfusová et al., 2021) provide a foundation for this work. Finally, a deeper mechanistic understanding of how environmental gradients—including precipitation, altitude, and temperature (Li et al., 2023a, b, 2024b)—shape SV is crucial for forecasting adaptive trajectories.

### Subgenome dynamics and genomic stabilization mechanisms

The stability of newly formed polyploid genomes is not automatic but is maintained through a dynamic equilibrium between genetic diversification and stabilization. This equilibrium is underpinned by molecular buffering systems that preserve genomic integrity. For example, genomic analyses in polyploid wheat show that centromeres restructure via distinct paths: the A subgenome experiences recurrent retrotransposon invasions, while the D subgenome undergoes gradual epigenetic expansion of CENH3 domains, collectively supporting centromeric rewiring (Huang et al., 2025). Nevertheless, direct functional evidence establishing causality in plants remains limited. Concurrently, comparative genomic assemblies reveal landscapes where lineage-specific retrotransposon proliferation co-occurs with SVs and subgenome divergence, a configuration that has been implicated in post-polyploid genomic stabilization (Shen et al., 2024). Thus, SVs provide raw material for adaptation, whereas mechanisms ensuring centromere function and modulated TE activity may buffer against excessive instability.

The physiological consequence of this balance is evident in traits like fertility. In *E. nutans*, high genome-wide heterozygosity correlates strongly with reduced fertility—this is particularly associated with SVs such as chromosomal rearrangements and pairing abnormalities in the more dynamic St and H subgenomes (Liu et al., 2022a, b). Another widespread outcome is subgenome dominance, a well-documented phenomenon in allopolyploids where one ancestral genome contributes disproportionately to the transcriptome. Critically, similar patterns of dominance and structural diversification are observed in diverse allopolyploids: wheat exhibits subgenome-specific centromere restructuring and dominance in chromosomal stability (Zhao et al., 2023), while cotton shows asymmetric expression and structural differentiation between A and D subgenomes (Rahman et al., 2021), suggesting common evolutionary principles.

However, the causal regulatory mechanisms orchestrating this balance remain incompletely understood. Key unresolved questions include how genetic differentiation among St/H subgenomes coordinates with adaptive traits (e.g., stress tolerance, fertility) in polyploid *Elymus* species, and the specific role of epigenetic regulation—a factor inferred to be critical for mediating environmental adaptation and genomic stability, yet its mechanistic links to observed genetic variation remain uncharacterized (Li et al., 2024b; Xiong et al., 2024a). Advancing this understanding requires moving from these correlative observations to causal inference. Future work should employ spatiotemporal functional genomics approaches such as scRNA-seq to dissect subgenome-specific expression dynamics, and chromatin immunoprecipitation sequencing (ChIP-seq) to map chromatin states across diverse environmental conditions. These approaches must be coupled with functional validation, for example, using CRISPR-based editing to perturb specific candidates like subgenome-specific transcription factors or TE families linked to adaptation. Ultimately, integrating epigenomic analyses will be essential to delineate how these epigenetic marks directly mediate St/H subgenome dynamics and long-term genomic stabilization in *Elymus*.

### Translating genomic dynamics into crop breeding

The study of dynamic subgenome interactions in polyploid plants provides a framework for addressing complex trait architecture, such as the “missing heritability” problem in crop breeding. Insights from these systems suggest several concrete translational strategies, grounded in empirical studies, which are summarized in **Table 1**.

**Table 1:** Breeding strategies informed by the *Elymus* negotiated genome.

Pathway	Objective	Key example/approach	Current challenge/future research need	Translational gap
Bridging heritability gaps	Introgress adaptive alleles from wild relatives	1. Leaf rust resistance via *E. sibiricus* 3St chromosome introgression into wheat (Motsnyi et al., 2024); 2. Cold-tolerant germplasm 20HSC-Z9 (Liu et al., 2024); 3. Drought tolerance in *E. borianus/E. russelli* (Khan et al., 2022)	Linkage drag complicates precise allele transfer; complex genetic architecture of adaptive traits.	Separating desirable alleles from deleterious genomic backgrounds.
Enabling precision germplasm management	Guide conservation and breeding via genomic resources	1. *E. sibiricus* high-quality chromosome-scale reference genome (Shen et al., 2024); 2. Wheat-*E. sibiricus* introgression line characterization (Motsnyi et al., 2024); 3. KASP-based SNP fingerprinting for core collections (Li et al., 2025b); 4. Conservation of endemic taxa (e.g., *E. magellanicus*) (Wu et al., 2022; Chen et al., 2023b) and adaptive hotspots (Li et al., 2024b)	Limited high-quality genomic references for diverse *Elymus* taxa; unclear genetic boundaries of adaptive hotspots.	Connecting genomic data directly to breeding decision-making and conservation prioritization.
Refining predictive models	Improve genomic selection (GS) accuracy	Integrate GWAS-identified hub genes (e.g., TOPLESS) (Zhang et al., 2022b), TCP transcription factor regulatory networks (Liu et al., 2025), and key agronomic traits (Liu et al., 2022c) into GS models	GS models overlook SVs and epigenetic effects; limited integration of regulatory network information.	Translating molecular network insights into actionable markers for GS model optimization.
Accelerating *de novo* innovation	Create novel trait combinations via hybridization	1.Natural hybrid zones (e.g., *E. hispidus*×*E. repens*) as natural sources of traits (Urfusová et al., 2024); 2. Salt tolerance selection in synthetic wheat-*E. farctus* hybrids (Babosha et al., 2024)	Unpredictable outcomes of genomic interactions in hybrids; difficulty minimizing linkage drag in synthetic crosses.	Developing tools to predict favorable trait combinations from hybrid genomic negotiations.

This table outlines four key breeding strategies grounded in the genomic dynamics of *Elymus* (e.g., adaptive structural variation, subgenome dynamics). It details their objectives towards climate-resilient crop improvement, supported by empirical examples, and highlights the prevailing translational challenges.

Bridging heritability gaps. Wild relatives are key reservoirs of adaptive genetic variation. Successful examples in *E. sibiricus* include the introgression of a 3St chromosome into wheat via disomic addition lines, conferring robust leaf rust resistance at the adult stage (Motsnyi et al., 2024), and the identification of the elite cold-tolerant germplasm 20HSC-Z9 with 100% green-up rate after overwintering at -30°C (Liu et al., 2024). Systematic phenotyping under abiotic stresses can pinpoint further sources of resilience, such as drought tolerance in the Central Asian species *E. borianus* and *E. russelli*—these species exhibit enhanced proline accumulation and antioxidant enzyme activity under water deficit (Khan et al., 2022). Together, these cases validate the feasibility of a practical approach: utilizing the *Elymus* gene pool for adaptive traits through targeted phenotyping and chromosome-mediated introgression.

Enabling precision germplasm management. High-quality chromosome-scale reference genomes of *E. sibiricus* (Shen et al., 2024) and molecular characterization of wheat-*E. sibiricus* introgression lines (Motsnyi et al., 2024), combined with genome-wide single nucleotide polymorphism (SNP) fingerprinting via Kompetitive Allele-Specific PCR (KASP) technology (Li et al., 2025b), empower gene discovery and facilitate the assembly of an optimized core collection that captures maximal adaptive diversity in *E. sibiricus*. This molecular information critically informs the conservation of endemic *Elymus* taxa including the South American endemic *E. magellanicus* (studied using cultivated accessions in China) (Wu et al., 2022; Chen et al., 2023b) and the Chinese endemic *E. breviaristatus* complex (Tan et al., 2022) as well as the delineation and protection of adaptive hotspots such as the Mekong-Salween Divide in QTP *Elymus* populations (Li et al., 2024b) and genetic structuring of *Elymus sensu stricto* on a global scale (Leo et al., 2025).

Refining predictive breeding models. For instance, in *E. sibiricus*, this process involves elucidating the genetic architecture and integrating core elements into genomic selection (GS) models to prioritize functional variants: genome-wide association studies (GWAS)-identified hub genes (e.g., TOPLESS) that regulate auxin/jasmonic acid (JA) signaling and plant height (Zhang et al., 2022b), critical regulatory networks (e.g., TCP transcription factors) linked to tillering capacity and stress-adaptive phenotypes (Liu et al., 2025), and key agronomic traits (e.g., spike architecture traits including spike weight and grain density) (Liu et al., 2022c). This integrated strategy enhances the predictive accuracy for target traits, advancing predictive breeding of high-yield and stress-tolerant *Elymus* cultivars.

Accelerating *de novo* trait assembly. Natural hybrid zones serve as natural sources of novel trait combinations, generating diverse genotypes via successive introgression and polyploidization (Urfusová et al., 2024). Similarly, synthetic wide hybrids serve as valuable platforms for trait innovation. For instance, in Russia, synthetic wheatgrass-*E. farctus* hybrids (derived from ×*Trititrigia cziczinii* × *E. farctus* crossed with the wheat-wheatgrass hybrid w107) utilize leaf micromorphological traits (e.g., silicified wavy-walled long cells, shield-shaped prickles) as selectable markers to track genomic introgression, facilitating the identification of desirable wild alleles (e.g., *E. farctus*-derived salt tolerance) while minimizing linkage drag of undesirable traits (Babosha et al., 2024). Together, these natural and synthetic hybrid systems streamline the assembly of *de novo* traits by leveraging genetic novelty from hybridization and polyploidization, accelerating the development of improved cultivars.

Remaining challenges and future research priorities. Significant hurdles persist, including linkage drag during introgression and limited accuracy of GS models that overlook SVs and epigenetic effects. Future research should focus on three key areas: first, targeted introgression strategies combining precise tools like CRISPR with insights into meiotic stability (Liu et al., 2022b) to enhance allele transfer efficiency; second, next-generation GS models integrating pan-genome data (Bayer et al., 2020), epigenetic markers (Amin et al., 2025), and species-specific regulatory networks (Zhang et al., 2022b; Liu et al., 2025); finally, advanced predictive frameworks synthesizing environmental associations (Xiong et al., 2022a; Li et al., 2023b) and hybrid zone dynamics (Urfusová et al., 2024) to guide resilient crop genotype design.

## A modular toolkit for stress resilience

Plant resilience to environmental stress is governed by a complex, modular system of signaling pathways and regulatory components (Zhu, 2016). Species of *Elymus*, which thrive in extreme habitats like the QTP, serve as a robust model for this modularity. Their adaptation is underpinned by the integrated function of these discrete modules. At the whole-plant level, this is illustrated by wild *E. nutans* populations in Tibet, where environmental gradients shape phenotypic variation and seed element profiles (Long et al., 2025). Decoding this modular architecture in *Elymus* provides a valuable blueprint for engineering climate-resilient crops. Core elements of this system include conserved pathways such as the C-repeat binding factor (CBF) regulon (Kopeć et al., 2022), symbiotic microbial networks (Zhu et al., 2022), and epigenetic regulators (Zhang and Zhu, 2025), which operate both independently and in coordination.

### Core molecular pathways

The resilience of *Elymus* species relies on a series of discrete molecular modules, each adapted to particular environmental challenges, as seen in the following key pathways: In response to cold, melatonin cooperates with the transcription factor WRKY11 to enhance antioxidant defense (Zhuoma et al., 2024). Drought triggers distinct molecular strategies across species. For *E. sibiricus*, this involves an integrated drought-response program: ABA signaling is turned on through up-regulation of EsSnRK2, EsLRK10, and EsCIPK5; the cuticle is reinforced by EsCER1-dependent synthesis of very-long-chain alkanes; and root suberin deposition is increased via induction of CYP86A1 and KCS20 (Liu et al., 2022d; An et al., 2024). In *E. nutans*, drought induces ABA accumulation, an early signaling event that likely primes subsequent adaptive pathways (Long et al., 2024). When exposed to combined cold and drought stress, the CBF regulon acts as a master transcriptional regulator, redirecting carbon toward osmoprotectants such as soluble sugars and proline (Liu et al., 2023b).

Salt tolerance in *Elymus* depends on specialized molecular programs. Tolerant *E. sibiricus* accessions regulate ion transporters to maintain a low Na^+^/K^+^ ratio, upregulate proline synthesis genes for water retention, and enhance antioxidant enzyme expression to limit electrolyte leakage (Chen et al., 2023a). In *E. nutans*, the molecular response to salt is distinct from its drought adaptation; salt stress activates ion-balance pathways, reflecting distinct stress-specific regulatory networks (Long et al., 2023). Heavy-metal detoxification in *Elymus* involves a dual strategy: antioxidant systems are transcriptionally activated alongside the balanced expression of anti-apoptotic (BI-1, UCP1) and pro-apoptotic (VPE, MYB391) genes to modulate programmed cell death. In this process, reactive oxygen species act as a critical signal (Hu et al., 2023; Han et al., 2024). A consistent pattern emerging from these studies is a dose-dependent transcriptional shift: growth-related genes (e.g., for photosynthetic pigments) are favored under mild stress, while defense pathways dominate under severe stress (Hu et al., 2023).

Beyond these specialized stress-specific modules, the resilience of *Elymus* also relies on higher-order regulatory networks that integrate signals across different stress modules. This integration occurs through central hubs. For instance, the transcription factor EsiERF285 fine-tunes drought and heat tolerance by coordinating the aforementioned ABA and antioxidant signaling pathways (Xiong et al., 2024b). Multi-omics studies identify other integrators, such as SAPK3, which sequentially activates hormone pathways under salt stress, thereby balancing growth and defense (De et al., 2024). Such integrated networks further influence life-history traits and resource allocation, directing metabolic flux toward synthesis of protective compounds like vitexin and allantoin under stress (Yu et al., 2022; Zhang et al., 2024a).

Modularity also applies to developmental programs, not just acute stress responses: Flowering time in *E. sibiricus* is controlled by modular networks that integrate environmental signals such as drought and salinity (Zheng et al., 2022). Similarly, during seed development, co-expression networks and hub genes (e.g., NAC, AP2/ERF) regulate starch metabolism (Zheng et al., 2024), effectively embedding resilience into the plant’s lifecycle.

Key future challenges include deciphering how these modules interact under combined stresses and evaluating the risks of engineering core trade-offs. Future research should characterize inter-module connectivity using multi-omics approaches and develop context-sensitive genetic switches—for example, via CRISPR—to activate specific adaptive modules only when needed. Understanding these dynamics, including the dose-dependent and developmental regulation outlined above, will be crucial for designing resilient crops.

### The synergistic microbial module

The rhizosphere microbiome is integral to the stress resilience of *Elymus* species, mediating key beneficial interactions. For example, colonization by arbuscular mycorrhizal fungi (AMF) primes plant defense via JA signaling and volatile organic compounds (VOCs) induction (Zhang et al., 2022a) and reprograms host metabolism under stress, upregulating flavonoid and lipid biosynthesis to enhance cold resistance (Zhang et al., 2023). Moreover, AMF reduces arsenic (As) uptake and translocation, thereby improving arsenic sequestration in *E. sibiricus* (Gatasheh et al., 2024). Similarly, fungal endophytes like *Epichloë* offer direct chemical defense through alkaloids (Du et al., 2024) and enhance cadmium (Cd) tolerance in *E. dahuricus* by bolstering antioxidant capacity and sustaining growth (Shi et al., 2024).

Notably, the assembly and function of this beneficial microbiome are themselves modulated by environmental factors. In *E. nutans* silage, for example, altitudinal gradients restructure the microbial community—selecting for high-elevation adapted lactic acid bacteria (e.g., *Lactiplantibacillus*, *Pediococcus*) while improving fermentation quality (Li et al., 2024a; Su et al., 2024).

Beyond these microbial synergies, the resilience toolkit of *Elymus* extends to perceiving chemical cues from its plant neighbors. Exposure to allelochemicals from *Artemisia baimaensis* (via litter leachates or VOCs) primes *E. nutans*, activating its antioxidant enzyme systems (e.g., SOD, POD, APX) and osmotic adjustment mechanisms (e.g., soluble sugar, proline accumulation) (Yang et al., 2023). This indicates that interplant signaling can be a supplementary layer of resilience.

To harness this synergistic potential for practical agroecological use, key challenges must be addressed. These include ensuring the functional stability of introduced microbial consortia across heterogeneous soil environments and understanding their compatibility with the plant’s endogenous signaling networks. Future efforts should therefore focus on two complementary strategies: first, the rational design of stable, environment-adapted synthetic microbial consortia (SMCs) that combine complementary taxa like AMF and *Epichloë*; and second, the targeted engineering of root exudate profiles (e.g., via CRISPR/Cas) to modulate the rhizosphere microbiome. Together, these approaches aim to purposefully strengthen this external biological defense barrier against abiotic stress.

### The epigenetic regulatory layer

Epigenetic mechanisms, such as dynamic DNA methylation, serve as critical fine-tuners of modular stress responses, underpinning phenotypic plasticity and the establishment of stress memory (Zhu et al., 2025). In plants, this regulation is bidirectional; for instance, active demethylation at defense gene loci can release transcriptional repression to potentiate immunity (Zhu et al., 2025), while precise methylation patterns are indispensable for processes like seed development (Frost et al., 2024). These epigenetic states often exert their phenotypic effects by governing downstream transcriptional regulators. A compelling illustration in *Elymus* is the targeted editing of the transcription factor EnTCP4 in *E. nutans* (Li et al., 2025a). Its disruption concurrently delayed flowering and enhanced drought tolerance—the latter linked to increased trichome density and superior water retention. This example underscores how modifying central regulatory nodes can rebalance the trade-offs between development and stress resilience, offering a strategic pathway for *de novo* domestication and molecular design breeding.

To advance from correlation to mechanistic understanding and application, future work should focus on three aims: establishing causal links via targeted epigenetic editing tools such as dCas9-effector fusions (deactivated Cas9 fused with epigenetic modifier domains) (Jogam et al., 2022); developing predictive panels of epigenetic markers to evaluate adaptive potential (Spadafora, 2023); and validating combined priming strategies under realistic field conditions.

Research on *Elymus* species converges on a modular view of stress resilience, wherein core molecular pathways, symbiotic microbial functions, and epigenetic regulation constitute a toolkit of complementary mechanisms. This understanding not only elucidates the architecture of adaptation but also provides an actionable framework for trait stacking in crop improvement. Thus, the path forward lies in the predictive assembly of these modules, marking a shift from protecting crops against stress to engineering inherent resilience.

## *Elymus* as an ecosystem engineer

Beyond its value as a genetic resource, *Elymus* also functions as an ecosystem engineer, contributing to sustainable agroecosystems through carbon sequestration, soil restoration, microbiome-mediated services, and invasive species suppression. The synergy between these multifunctional traits with technologies like microbiome engineering and remote sensing (Wang et al., 2024) further enhances its utility for rehabilitating degraded lands.

### Engineering ecosystem services

A central service provided by *Elymus* is the improvement of soil structure and stability. For instance, when grown with biochar amendments, *Elymus* grasses significantly boost soil organic carbon content—highest by 222% compared to controls—thereby reinforcing soil health and carbon sequestration potential (Ashraf and Chen, 2023). In erosion control, *E. tangutorum* works synergistically with *Poa pratensis* to enhance litter interception and rainfall infiltration, reducing soil erosion by 65% on slopes of the QTP (Liu et al., 2022e).

Beyond soil improvements, its performance can be enhanced through targeted management. Under precision interventions, the co-application of copper nanoparticles (CuNPs) and AMF alleviates As stress in *E. sibiricus* by reducing As uptake and strengthening antioxidant enzyme activity (Gatasheh et al., 2024). Similarly, nano-Fe_3_O_4_ application mitigates Cd toxicity by regulating anti-apoptotic genes and suppressing pro-apoptotic genes, thereby sustaining plant biomass (Han et al., 2024).

*Elymus* also actively shapes biotic communities and suppresses stressors. Reseeding of *E. nutans* in the alpine meadows of eastern QTP modifies habitat structure, suppressing populations of the plateau pika (*Ochotona curzoniae*) (Wei et al., 2022). Additionally, fungal endophytes from European wild *E. repens* exhibit biocontrol activity against plant pathogens including *Fusarium culmorum* and *Pyrenophora teres*, adding another layer of defense against biotic threats (Høyer et al., 2022). Its competitive advantage in light capture enables *E. canadensis* and *E. virginicus* to suppress the invasive common buckthorn (*Rhamnus cathartica*) in the deciduous forest understories of Minnesota, USA, further stabilizing plant community composition (Schuster et al., 2025).

However, translating these benefits into reliable, large-scale applications faces challenges. These include ensuring the long-term stability of managed soils and navigating ecological trade-offs. For instance, while legume-*Elymus* mixtures improve soil fertility, they may increase the abundance of soil pathogenic fungi under warming conditions in field experiments (Zhong et al., 2023). Monocultures risk autotoxicity, as demonstrated in *E. sibiricus*, where rhizosphere soil extracts inhibit the growth of conspecific seedlings in laboratory assays (Yang et al., 2022). Additionally, allelopathic interference from invasive species, such as the inhibition of *E. nutans* germination and growth by the toxic grass *Achnatherum inebrians*, as observed in greenhouse studies (Zhang et al., 2024b), presents another concern.

### Engineering the rhizosphere microbiome

In *Elymus* species, the rhizosphere microbiome constitutes a stable, functionally integrated consortium that underpins host adaptation and ecosystem resilience. This consortium exhibits multigenerational stability; for instance, in *E. nutans*, key functional groups like ammonia-oxidizing bacteria (e.g., *Nitrosospira multiformis*, *Nitrosospira* sp. *Nsp17*) are vertically transmitted, thereby ensuring the persistence of nitrogen (N)-cycling functions (Liang and Bowatte, 2022). In *E. dahuricus*, the host plant actively modulates its rhizosphere consortium through root exudates and associated organic matter, selectively enriching microbes that enhance both soil fertility and plant stress tolerance (Hao et al., 2023).

The rhizosphere system of *Elymus* exhibits strong functional synergy among its microbial partners. Plant growth-promoting rhizobacteria (PGPR), such as *Serratia* and *Bacillus* strains, enhance root architecture and nutrient uptake under cold stress (Ran et al., 2025). Meanwhile, AMF like *Funneliformis mosseae* and *Rhizophagus irregularis* contribute to metal sequestration, cold tolerance, and the systemic priming of plant defenses (Zhang et al., 2022a, 2023; Gatasheh et al., 2024). A notable example of this synergy is seen in *E. nutans*, where AMF colonization establishes a positive feedback loop: improved P and N uptake boosts plant biomass, which in turn reinforces a systemically primed defense against herbivores like *Locusta migratoria* through enhanced JA signaling and the production of insect-deterrent volatiles (Zhang et al., 2022a).

To harness this functional synergy for agricultural applications, a central challenge lies in translating this knowledge into robust practices, particularly in maintaining the field stability of SMCs (Sharma and Araujo, 2025) and mitigating potential ecological risks, such as the unintended enrichment of pathogens in managed systems (Zhong et al., 2023). Future work should aim to design context-specific SMCs by leveraging metagenomic data and predictive modeling, and concurrently establish robust ecological risk assessment frameworks to guide the responsible deployment and monitoring of engineered microbiomes.

### Precision ecosystem management

Precision management of *Elymus*-based ecosystems begins with optimized sowing strategies. Spatial arrangement is a key factor: cross-row monoculture planting of *E. nutans* increases yield by reducing intraspecific competition, while mixtures with legumes such as *Onobrychis viciifolia* enhance overall community biomass and improve lodging resistance compared to monocultures (Ren et al., 2023). Designing sowing patterns including square-sowing to balance these inter- and intraspecific interactions is therefore essential for stable productivity (Ren et al., 2025).

Subsequent management interventions, such as mowing, can precisely direct plant development. In *Elymus* species, mowing at a defined intensity and timing, for instance moderate mowing at the jointing stage, promotes biomass allocation to roots and stimulates compensatory growth, with root biomass serving as a major predictor of regrowth capacity (Yang et al., 2025).

These principles can be extended through monitoring and targeted control technologies. For beneficial *Elymus* species in China, this includes using species distribution models to identify optimal planting areas in the northwest and western mountainous regions (Lu et al., 2025a). It also involves targeted chemical approaches—such as combining broad-leaved grass inhibitors with nitrogen fertilization—to boost seed yield and suppress weeds in *E. nutans* stands on the QTP (Lu et al., 2025b).

For *E. repens*, however, a differentiated management strategy is needed, especially in Northern Europe. Management planning must first account for its invasive potential—a critical factor to consider in introduction plans (Ringselle et al., 2023). For its control, sustainable non-chemical methods like low-energy lasers or electrical treatments are practical options (Andreasen et al., 2024; Ringselle et al., 2025). Additionally, understanding its dose-dependent allelopathy can refine control strategies: studies in Ukraine show that low concentrations of its rhizome extracts stimulate wheat growth, while high concentrations inhibit it (Okrushko, 2022).

Translating ecological research into practice involves addressing scale-up challenges. For instance, 3D-printed *Elymus* experimental surrogates—which mimic key biomechanical traits—address the problems of seasonal variability in plant traits and sample standardization in field studies, serving as a critical bridge between controlled experiments and real-world application (Keimer et al., 2024). However, the economic feasibility of deploying such tools at the field scale remains a major hurdle. Furthermore, management practices such as mowing and fertilization require careful balancing of short-term yield against long-term stand persistence—excessive mowing or fertilization can undermine persistence, whereas optimized regimes (e.g., moderate mowing at the jointing stage, balanced nutrient input) support sustained productivity (Wu et al., 2024; Yang et al., 2025). Precision nutrient management, using site-adapted optimized nitrogen-phosphorus-potassium (N-P-K) formulations, is therefore essential to maximize both economic returns and ecological sustainability (Song et al., 2025).

Future efforts should focus on integrating these technological approaches into cohesive farm management systems. Key implementation pathways include: developing Internet of Things (IoT)-enabled autonomous equipment capable of synchronized operations such as weeding and seeding, supported by real-time mechanical and environmental sensing (Rajak et al., 2023); establishing integrated monitoring platforms that combine unmanned aerial vehicle (UAV)-based multispectral remote sensing for high-throughput disease phenotyping and yield prediction (Chivasa et al., 2021) with pervasive IoT sensor networks for real-time surveillance of soil conditions, microclimate, and pest dynamics; and applying predictive modeling tools to dynamically optimize target species productivity and environmental sustainability through data synthesis and intelligent decision support.

Building on *Elymus*’ role as an ecosystem engineer, an integrated framework can be designed to balance productivity with resilience. This approach synthesizes precision agronomy, rhizosphere microbiome engineering, and smart monitoring technologies (e.g., IoT and UAV-based remote sensing). It enables applications such as precision restoration units—combining selected seeds, tailored microbial consortia, and amended growth substrates—and adaptive management systems driven by real-time sensor data. Implementing these tools at scale, however, requires overcoming key challenges: ensuring cost-effectiveness, maintaining long-term soil and microbiome stability, and managing ecological risks such as species trade-offs or autotoxicity.

## Conclusion

**Figure 1:**
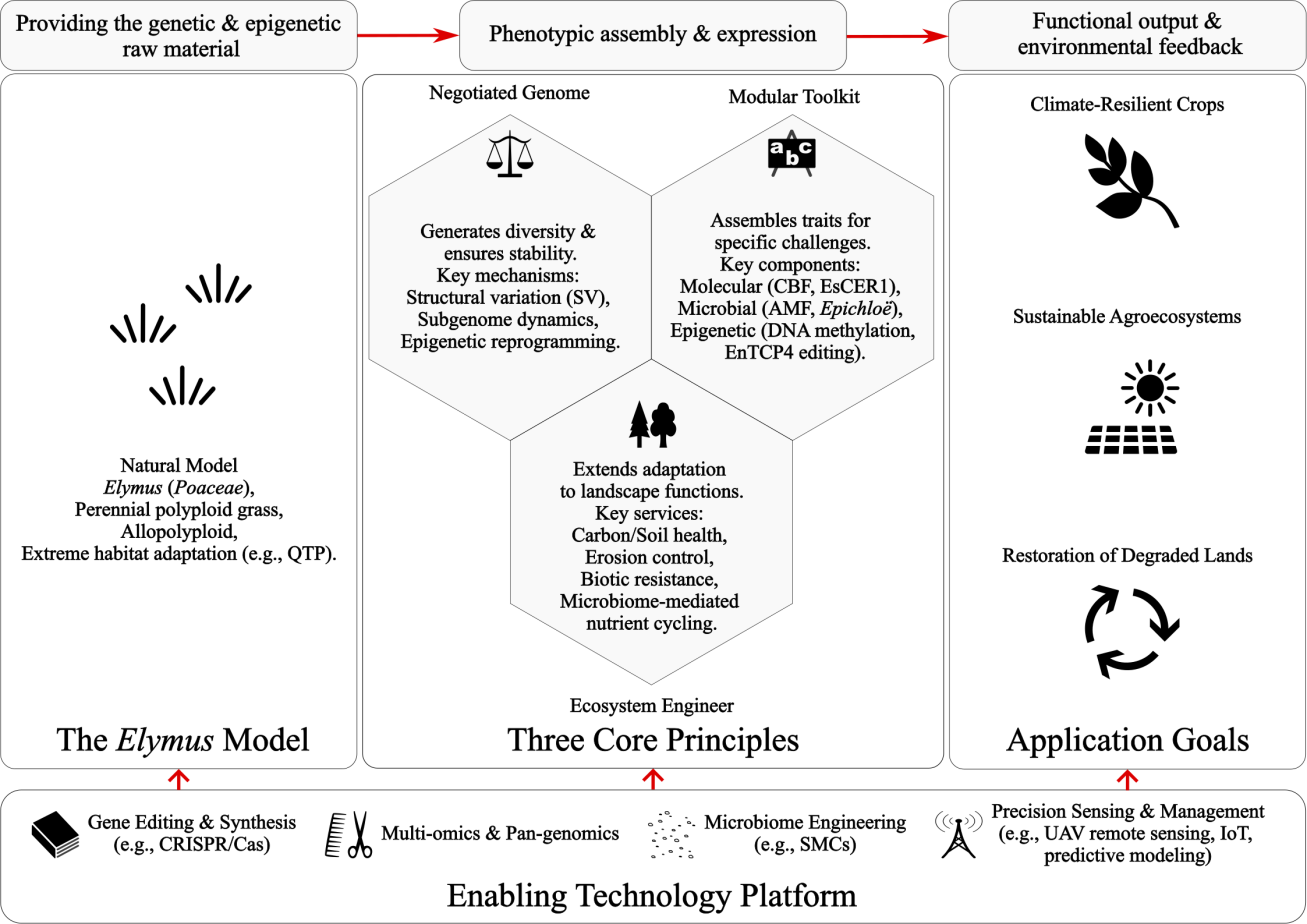
Conceptual framework of the *Elymus* Model for climate-resilient crop and agroecosystem design. The model is derived from *Elymus* (central left), a perennial allopolyploid grass that thrives in extreme habitats including the QTP, following a left-to-right translational flow. Its adaptive success rests on three core interconnected principles: a dynamically negotiated allopolyploid genome (generating diversity through SV, subgenome dynamics, and epigenetic reprogramming), a modular toolkit (assembling molecular, microbial, and epigenetic components for specific stresses), and ecosystem engineering (delivering landscape-level services including carbon sequestration, erosion control, and microbiome-mediated nutrient cycling). These principles are enabled by a cross-cutting technology platform (bottom) that includes gene editing, multi-omics, microbiome engineering, precision sensing, and predictive modeling. The integrated framework guides the development of climate-resilient crops and the restoration of degraded lands toward sustainable, multifunctional agroecosystems (central right).

The *Elymus* system offers a comprehensive framework for understanding and engineering climate-resilient plants—what we term the *Elymus* Model. See **Figure 1** for a visual synthesis of this framework, which integrates three evolutionarily aligned pillars: (1) a dynamically negotiated allopolyploid genome that balances structural innovation with meiotic stability; (2) a modular toolkit comprising molecular, microbial, and epigenetic components, which can be combined to counter specific stresses; and (3) keystone ecosystem functions that extend adaptive benefits from individual plants to the landscape scale.

A key distinction between the *Elymus* Model and adaptation strategies in other plant systems lies in its deeply integrated, multi-scale resilience. For instance, while suberin deposition is a common drought response across plants, *Elymus* species like *E. sibiricus* coordinate suberin biosynthesis through a network of subgenome-tuned regulators (e.g., EsCER1, CYP86A1, KCS20) (Liu et al., 2022d; An et al., 2024). This reflects a genome-level capacity—rooted in subgenome negotiation—to fine-tune universal adaptive traits, setting it apart from simpler stress response pathways in other taxa like *Arabidopsis thaliana* and *Oryza sativa* (Zhu, 2016). Similarly, its modular stress toolkit unites hormone signaling, microbiome recruitment, and epigenetic memory as a coordinated system, rather than a collection of isolated pathways.

By unpacking how polyploidy, modularity, and ecosystem engineering interact in *Elymus*, this model provides more than a crop improvement blueprint. It offers a framework for designing inherently adaptive agroecosystems. Insights from *Elymus* underscore that sustainable agricultural innovation can be powerfully guided by evolutionary strategy—moving beyond mere trait transfer toward the principled design of resilient crops and landscapes.

## Future perspectives

The *Elymus* Model establishes a concrete framework to guide future research and translation. Future work should focus on bridging key knowledge gaps and converting systemic insights into practical applications.

Resolving mechanistic uncertainties. A primary challenge is to move from correlation to causation across the model’s pillars. For the negotiated genome, functional validation is needed to show how subgenome communication and epigenetic reprogramming directly orchestrate adaptation—going beyond the well-documented role of SV. Within the modular toolkit, research must elucidate the connections between molecular, microbial, and epigenetic components, especially under concurrent stresses. Expanding phylogenomic studies to underrepresented *Elymus* species globally will clarify how hybridization and polyploidization histories shape these adaptive architectures.

Translating systemic insights into breeding and management. The model’s value lies in its application. In breeding, this requires next-generation prediction models that integrate pan-genomic structural variants, epigenetic marks, and regulatory network data to capture complex resilience traits. For agroecosystem design, the principles of modularity and ecosystem engineering call for practical tools—such as decision-support frameworks for deploying *Elymus*-based mixtures in degraded lands (e.g., site-specific seed-ratio recommendations or matched microbial consortia) or for engineering root-exudate profiles to steer beneficial microbiomes. Crucially, the deployment of novel genotypes or synthetic microbial consortia must be coupled with rigorous, long-term ecological risk assessment to monitor outcomes such as soil pathogen dynamics or community stability.

Addressing scalability and trade-offs. Implementing the model at scale faces practical hurdles: the cost and robustness of precision-management technologies, the persistence of engineered traits or microbiomes in variable field environments, and the management of ecological trade-offs (e.g., between productivity and stand persistence, or between species in mixtures). Overcoming these barriers requires not only technical innovation but also a shift in agroecosystem design philosophy—from optimizing single crops to managing resilient, multifunctional systems.

In summary, *Elymus* transcends its role as a model genus; it provides an evolutionary blueprint for rethinking agricultural resilience. By integrating its principles with advances in genetics, microbiology, and precision ecology, we can develop agricultural systems that are inherently more adaptive and sustainable. The path forward demands a concerted effort to ground this blueprint in mechanistic understanding and to test its application in the complex reality of working landscapes.
